# Harvesting time and roasting effects on colour properties, xanthophylls, phytates, tannins and vitamin C contents of orange maize hybrid

**DOI:** 10.1038/s41598-020-78433-9

**Published:** 2020-12-07

**Authors:** Emmanuel O. Alamu, Busie Maziya-Dixon, Abebe Menkir, Adebayo O. Ogunlade, Olorunfemi Olaofe

**Affiliations:** 1 Food and Nutrition Sciences Laboratory, International Institute of Tropical Agriculture, Southern Africa Research and Administration Hub (SARAH), Campus, PO Box 310142, 10101 Chelstone, Lusaka Zambia; 2 International Institute of Tropical Agriculture (IITA), PMB 5320, Oyo Road, Ibadan, Oyo State Nigeria; 3 Center for Healthful Behavior Change, Department of Population Health, New York University Grossman School of Medicine, New York, NY USA; 4 Department of Chemistry, Ekiti State University, Ekiti State, P.M.B.5363, Ado-Ekiti, Nigeria; 5 International Institute of Tropical Agriculture (IITA), 7th Floor, Grosvenor House, 125 High Street, Croydon, CRO 9XP England, UK

**Keywords:** Biochemistry, Chemical biology, Plant sciences, Environmental sciences, Chemistry

## Abstract

Biofortified maize varieties form an essential part of a nutritious diet; available evidence suggests that different processing methods may affect the final food products. The study aimed to evaluate the effects of processing (roasting) and harvesting time on the bioactive components (lutein, zeaxanthin, β-cryptoxanthin, phytate, tannin and vitamin C) and colour properties (L*, a*, b*), of biofortified orange maize. The orange maize hybrids used for the study were obtained from the International Institute of Tropical Agriculture (IITA) diverse lines with high provitamin A (PVA) content. The results showed that harvesting time and roasting methods significantly (P ≤ 0.001) affected the colour properties. The positive values of ∆b* 30.7, 36.0 and 38.1 at 20 days after pollination (DAP), 27DAP and 34DAP, respectively showed that the intensity of orange colour increased with delay in harvesting time. In unprocessed freshly harvested orange hybrid maize; lutein, zeaxanthin, β-cryptoxanthin, tannin and vitamin C increased with an increase in harvesting time. For roasted hybrid, the mean concentrations of all the bioactive components increased with increases in harvesting time except for tannin and vitamin C that showed a decrease at 20DAP and 27DAP. The results revealed that processing and time of harvest affect the levels of non-provitamin A carotenoids, tannins, phytic acid, Vitamin C and the colour properties of biofortified maize genotypes.

## Introduction

Maize (*Zea mays *L.) is an important cereal crop in the world and an essential staple in the diets of millions of households in sub-Saharan Africa and Latin America^1^. According to the Food and Agriculture Organization estimate, more than 125 million metrics tons of maize were produced for human consumption in 2013, an increase of about 7.5 million metric tons from the 2010 estimate^2^. Although, the most well-known and widely grown varieties in the tropics are white and orange maize; several human efficacy trials have shown that the consumption of staple crops biofortified with micronutrients can alleviate micronutrient deficiencies among nutritionally at-risk population groups^3,4^. Green maize (freshly harvested maize) is consumed widely in Nigeria and other parts of Africa in its season, and its utilization includes boiled and roasted green maize. Biofortification promises to be a viable strategy for improving the micronutrient status of millions of nutritionally at-risk population groups^5–7^. While biofortified maize varieties may form an essential part of a nutritious diet, available evidence suggests that different processing methods may affect the final food products. We previously showed the impact of processing methods on the provitamin A carotenoid composition of biofortified orange maize varieties^8,9^. However, there is little information on the effects of processing on the colour properties (an important attribute that decides consumer acceptability of the final food product) and non-provitamin A carotenoid, particularly lutein, zeaxanthin; as well as other vital bioactive components including tannins, phytic acid and ascorbic acid (all these bioactive constituents have essential health impacts). The pigment in the macula of the human retina is made up of lutein and zeaxanthin^10^. It was believed that they are responsible for the protective ophthalmologic effect of carotenoids, acting both as a filter of high-energy blue light and as an antioxidant. In a review published by Stahl et al.^11^ and Alves-Rodriguez and Shao^12^, lutein has been linked to the lowering of the risk for cataract development. Cataract extraction is one of the most frequently performed surgeries in the elderly. However, lutein occurs mainly outside the macular and significant carotenoid in the macular is zeaxanthin which exists in a trace amount in the typical diet but abundant in orange maize. Since maize is consumed mainly after being processed, thus most of these carotenoids are loss through the processing. The phytic acids occur as inositol hexaphosphate (IP6) in foods and have been reported to have the potential to act as an anticancer agent^13^. In whole-grain cereals such as maize, wheat and rice the ranges of phytic acid are from 1.5 to 6.4%, while defatted and dehulled oilseed meals such as soy, peanut and Sesame contain 1.5% or more of the compound^14^. The heat treatments effect on the phytic acid content of maize products was studied by Khan et al. ^15^, and they reported that fresh mature corn contains less phytic acid (1.71 g/kg) than dry corn (7.15–7.60 g/kg). The loss of phytic acid varies from 18.1 to 46.7% for fresh maize and from 11.5 to 52.6% for dry maize respectively among the heat treatments given. The relationship between protein and phytic acid contents during maize grain maturation was found to increase till the later stage of the grain maturity^16^. Maize is known to high in vitamin C content, and it has been reported to exceed those in the apple and pear but less than what is obtained in citrus fruits^17^. There are numerous types of phenolics found in nature, including tannins and some important ones are present in maize. Polyphenols and carotenoids could exert an essential role in preventive nutrition, but they are susceptible to high variation among cultivars and growth conditions. There is little information on the effects of processing and maturity on the bioactive contents (especially the xanthophylls, phytates, tannins and vitamin C) of roasted fresh maize when roasted with or without husk on the cobs. Therefore, this study evaluated the effect of processing (roasting with and without husk) and harvesting time on the colour properties and bioactive components of biofortified orange maize hybrids. Bioactive compounds oxidation and loss during the processing of foods are of great concern for food scientists, nutritionists, processors and consumers.

## Materials and methods

### Sources of orange maize

The orange maize hybrids with high provitamin A (PVA) carotenoid content used for the study were collected from the promising pipeline genotypes developed by the IITA Maize improvement breeding program (Supplementary information S1). Eight orange maize hybrids (including Obatampa II variety as control) were planted at Ibadan, Oyo State with GPS 7° 22′ N, 3° 58′ E, altitude 150 m, annual rainfall 1312 mm and annual temperatures 20.3–33.8 °C and Ikenne, Ogun State with GPS 10° 40′ N, 8° 77′ E, altitude 730 m, annual rainfall 4420 mm and annual temperatures 22.3–33.4 °C. The hybrids were planted during the rainy season in May for two seasons and grown under rainfed conditions in a randomized complete block design (RCBD) with three replications. There was no application of fertilizers or herbicides applied during the experiment. The weeding was done manually when necessary.

#### Field sampling

Kurilich and Juvik^18^ and Doehlert et al.^19^ reported that there is maximum dry weight accumulation in kernel development between 15 and 30 days after pollination (DAP) and between 25 and 30DAP respectively. Therefore, we chose the three fresh market maturity stages between 20 and 34DAP. Plants were randomly pre-labelled on the field for the three harvest maturity stages of 20, 27 and 34 days after pollination (DAP) for each hybrid. They were harvested at 08.00 hrs on the relevant dates and samples were collected into the mailing sacks and transferred to the laboratory for analysis^9,20^. Each of the hybrids (3 replications) was divided into three sets for chemical assays, roasted with intact husk (undehusked cobs) and roasted without husk (dehusked cobs). However, the selections and divisions of the samples were strictly randomized.

### Processing of freshly harvested orange maize

Two batches of fifteen (15) freshly harvested cobs of each hybrid (3 replications) were selected for processing. One batch was roasted with husk, and the other batch was roasted without husk using hot-charcoal burning on wire gauze according to the local practice as described in another study^9^. We recorded the time taken to roast each of the samples, and the average time of roasting was calculated, the roasting time varied with harvesting times for both forms of roasting. Dehusked cobs from the harvesting time 20DAP, 27DAP and 34DAP roasted at an average time of 15, 12 and 10mins respectively, while undehusked cobs from 20, 27 and 34DAP harvests roasted at an average time of 20, 15 and 10mins, respectively. All the harvested cobs were processed within 12 h of harvest. The unprocessed and processed orange maize cobs for each hybrid and from each harvest meant for chemical assays were carefully shelled, uniformly freeze-dried using Labconco Freezone 4.5L (at the temperature of − 54 °C and vacuum pressure of 0.45 mbar). The freeze-dried samples were milled using Laboratory mill 310 from PERTEN using sieve size 0.5 mm, packed in a dark sample polythene whirl-pack and stored at − 80 °C until analyzed for carotenoids.

### Colour determination

This was determined using the colour meter (Colour Tec PCMTM Colour Tec Associates, Inc., 28 Center Street, Clinton, NJ 08,809). The colour meter operates on the CIE (Commission Internationale de l’Eclairage) L^*^, a^*^, b^*^ colour scheme. The CIELAB colour space is in a cube form, and the L* axis runs from top to bottom. The maximum for L* is 100, which represents a perfect reflecting diffuser. The positive a* value is red and negative a* value is green. The positive b* value is yellow and the negative b* indicates blueness. Multiple measurements of several points on samples were made. The instrument was first standardized (L* = 93.24, a* = 00.96, b* = -02.75) with a Business Xerox 80 g/m^2^ white paper with 136 CIE whiteness D65. Milled samples (3.0 g) were put in a clean paper, and the colour meter was placed on the sample by allowing the sensor to touch the sample. The readings were taken directly for L^*^, a^*^, b^*^. The instrument display three-dimensional colour difference in uniform colour space coordinates. There are delta values associated with this colour scale. ∆L* ∆a* and ∆b* indicate how much a standard and sample differ from one another in L*, a*, and b*. However, + ∆L* value means the sample is lighter than the standard, + ∆a* value means the sample is redder than the standard, + ∆b* value means the sample is yellower than the standard.

### RP-HPLC analysis for lutein, zeaxanthin and β-cryptoxanthin

The adapted method of Howe and Tanumihardjo^21^ was employed to assess the samples for carotenoid composition and content, as described by Alamu et al*.*^8,9^. The extraction of carotenoid from dried maize (0.6 g) was done by adding ethanol (10 ml) containing 0.1% butylated hydroxyl toluene (BHT). The dried extract was reconstituted in 1 ml of methanol/dichloromethane (50:50 ml/ml), and 100µL aliquot was injected into the HPLC system for analyses of lutein, zeaxanthin and β-cryptoxanthin. The HPLC system (Water Corporation, Milford, MA) was used for carotenoids quantification. Solvent A consisted of methanol: water (92:8 ml/ml) with 10 mmol/L ammonium acetate and solvent B consisted of 100% methyl tertiary-butyl ether. The gradient elution at the rate of 1 ml/min was employed by using linear gradient; 29 min from 83 to 59% A, 6mins from 59 to 30% A, 1 min hold at 30% A, 4 min from 30 to 83% A and a 4 min hold at 83%. Chromatograms were generated at 450 nm, and identification of the carotenoids was determined using external standards method based on calibration curves from pure standards and with verification of absorption spectrum and co-elution with available authentic standards. Standards of the carotenoids were purchased from CaroteNature, GmbH (Lupsingen, Switzerland). Solvents were of HPLC grade.

#### Determination of ascorbic acid

Ascorbic acid (vitamin C) was determined by the method of the Association of Official Analytical Chemists (AOAC)^22^. Briefly, 50 mg of 2, 6-dichloroindophenol sodium salt dissolved in 50 ml water to which had been added 42 mg sodium bicarbonate. The mixture was shaken vigorously, diluted to 200 ml with water; filtered and stored in an amber bottle in the refrigerator. The dye was standardized daily by taking three 2 ml aliquots ascorbic acid standard solution. The maize sample (50 g) was homogenized for 3 min in 150 ml of aqueous metaphosphoric acid (3 g /100 ml) and 8 g/100 ml acetic acid (HPO_3_-HOAc) using a warring blender. The slurry was made up to 200 ml with the extracting solution and vacuum filtered using a Buchner funnel and Whatman no.1 filter paper to give the extract. The vitamin C content was determined by directly titrating 50 ml of the extract with dye solution, at the endpoint, the colour changed to rose pink that persisted for more than 5 s.

### Phytic acid analysis

The phytic analysis was done by mechanical shaking of the mixture of 5 g finely ground sample in 50 ml trichloroacetic acid (TCA) (3 g/100 g). The supernatant (10 ml) was transferred into a 40 ml conical centrifuge tube and added 4 ml Ferric chloride solution (2 mg Ferric iron per ml in TCA). The tube and its contents were heated in a boiling water bath for 45 min and followed by centrifugation at 3500 rpm for 15 min and the supernatant carefully decanted. The precipitate was washed twice by dispersing well in 25 ml of TCA (3 g/100 ml), heating in boiling water bath 5 min and centrifuging. The precipitate dispersed in 3 ml of 1.5 mol/L NaOH with mixing. The suspension was filtered hot, and the precipitate washed with 60 ml hot water. The filtrate was discarded, and the precipitate from the paper was dissolved with 40 ml hot 3.2 mol/L HNO_3_ into 100 ml volumetric flask. 5 ml aliquot was transferred to another 100 ml volumetric flask and diluted to 70 ml. Additionally, 20 ml 1.5 mol/L KSCN was added, and volume made to 100 ml. Absorbance was read (within 1 min) at 480 nm. The iron content was calculated from a Fe (NO_3_)_3_ standard curves, and the phytate phosphorus was calculated from the iron results assuming a 4:6 iron: phosphorus molecular ratio.

### Determination of Tannins

The method of Joslyn^23^ and Padmaja^24^ were adapted for the analysis of total extractable polyphenolics using Folin-Dennis reagents. Folin-Dennis reagent (2.5 ml) and 10 ml sodium carbonate (17 g/100 ml) were added to the mixture. The solution was made to mark with water, mixed well and left to stand for 20mins. Absorbance was read at 760 nm. About 0.75 ± 0.001 g ground sample was weighed into a 50 ml beaker containing 20 ml methanol (50 g /100 ml). The solution was covered with parafilm and placed in a water bath at 77–80 °C for 1 h. The extract was filtered into a 50 ml volumetric flask using aqueous methanol (50 g/100 ml) to rinse and made up to volume with water. The solution was mixed, and 1 ml extract pipetted into a 50 ml volumetric flask. A measure of 20 ml water, 2.5 ml Folin-Dennis reagent and 10 ml sodium carbonate (17 g /100 ml) were added to the mixture. The solution was made to mark with water and allowed to stand for 20 min. Absorbance was read at 760 nm.

### Statistical analysis

The analytical data were reported as mean ± standard deviation of at least duplicate independent extractions of samples from two locations and for two seasons. The analysis of variance (ANOVA) and descriptive statistics using the Statistical Analysis System (SAS) software package version 9.2. were carried out on the data^25^. Least significant difference (LSD) test was used for mean comparison.

## Results and discussion

### Effects of roasting and harvesting time on colour properties of fresh orange hybrid maize

The results of the colour properties of unprocessed fresh orange hybrid maize at different harvest maturity stages are shown in Table 1 and Supplementary information S2. There was a decrease in the mean values for L* while a* and b* values showed an increase across the harvest maturity stages. The positive values of ∆b* 24.5, 29.8 and 31.9 at 20DAP, 27DAP and 37DAP respectively showed that the intensity of orange colour increased with delay in harvesting time. The total colour difference (∆E*), which considers the difference between the L*, a*, and b* of the samples, increased with the delay in harvesting time. However, there was no significant difference between the mean value of ∆E* at 27DAP and 34DAP. Besides, the ∆H* values followed the same pattern as the ∆E*, and this implies the hueness increased as the maize matured. The ∆H* is the difference in the hue angle between the sample and standard as described in a polar coordinate system. The observed changes in the ∆E* and ∆H* values indicate that the colour of the hybrid varieties changes as the maize matured and could be due to the increase in the concentration of the pigment.

**Table 1:** Colour properties of fresh orange hybrid maize at different harvesting time across genotypes and locations (N = 576).

Harvesting time	Colour parametes^a^							
	L*	a*	b*	ΔL*	Δ a*	Δ b*	ΔE*	ΔH*
**Unprocessed**								
20DAP	80.2a	4.27b	30.7b	− 8.11a	3.42b	24.5b	26.2a	35.0a
27DAP	80.0a	5.87a	36.0a	− 8.29a	5.03a	29.8a	31.7b	42.7b
34DAP	79.7a	6.29a	38.1a	− 8.61a	5.45a	31.9a	33.6b	45.8b
**Roasted without husk**								
20DAP	71.5ab	4.13c	26.5b	− 16.8ab	3.29c	20.3b	26.7c	29.1a
27DAP	72.5a	6.42b	33.4a	− 15.8a	5.57b	27.2a	32.4b	39.3a
34DAP	70.0b	7.47a	34.1a	− 18.3b	6.62a	27.9a	34.5a	40.6a
**Roasted with husk**								
20DAP	72.1a	7.30a	39.3a	− 16.2a	6.46a	33.1a	39.4a	47.7a
27DAP	72.2a	6.15b	32.6b	− 16.0a	5.30b	26.4b	31.6b	38.0b
34DAP	69.1a	6.22b	32.2b	− 19.2a	5.37b	26.0b	32.6b	37.5b
P (location)	ns	ns	ns	ns	ns	ns	ns	ns
P (maturity)	ns	***	***	ns	***	***	***	***
P (method)	***	***	***	***	***	***	***	***
P(location × maturity × method)	***	***	***	***	***	***	***	***
Error	18.8	0.94	6.96	18.8	0.94	6.96	14.8	13.9

L* = Lightness, a* = redness, b* = yellowness, ∆L*, ∆a*, and ∆b* = indicate how much a standard and sample differ from one another in L*, a*, and b*, ΔE* = total colour difference, ΔH* = hueness.

Mean values with similar letters in the same column do not differ significantly (p > 0.05).

*N* total sample data points.

^a^All colour properties were determined in duplicate.

*, **, ***Significant at P ≤ 0.05, P ≤ 0.01 and P ≤ 0.001 respectively, *ns* not significant P > 0.05.

Table 1 and Supplementary information S3 and S4 showed results for colour properties of fresh orange hybrid maize roasted with and without husk, respectively. When the hybrids were roasted without husk, the L* mean value for 20DAP was not statistically different (p > 0.05) from 27 and 34DAP, but 27DAP and 34DAP were different. The observed pattern suggested that roasting without husk (dehusked) affected the degree of lightness of the maize varieties at later stages of maturity. There was no significant difference among L* mean values for hybrids roasted with husk at all harvest maturity stages. There was a decline in the L* values from 27 to 34DAP. The finding agrees with the results of Adegunwa et al.^26^, and they mentioned that the low-moisture content, denaturation of proteins, and the concentrated amount of oil particles embedded in protein matrix could be reasons of colour variation during roasting of Sesame. Kahyaoglu and Kaya ^27^ observed similar results. The a* values for roasted fresh orange hybrid maize without husk were significantly different at all stages. Besides, there was no significant difference at 27DAP and 34DAP for roasted fresh orange hybrid maize with husk.

Kahyaoglu and Kaya ^27^ described that the higher roasting temperature and longer exposure time resulted in the more significant a* value and darker colour for a similar product. Makinde et al.^28^ also reported the same result of increased in the a* values of roasted Sesame seeds and stated that they were due to the formation of brown pigments through the non-enzymatic browning and phospholipids degradation. An increase in a* indicated a tendency to browning^29^, which might be attributed to the colour of reaction products from Maillard reaction and caramelization^30^. Chung et al.^31^ reported similar findings of corn kernels, but Ozdemir and Devres^32^ conversely observed a decline in the a* value of hazelnuts during roasting. However, there were higher values of L*, b*, and ∆E* for roasted hybrid maize with husk than values for roasted without husk across the three stages of harvesting. This observation suggested that husk has a positive effect on the colour properties of roasted fresh orange hybrid maize. There was also a general decrease in L*, b* and ∆E* values for roasted maize samples when compared with unprocessed maize samples. This present observation inferred that forms of roasting affect the colour properties of fresh orange maize. These properties could play a significant role in overall acceptability of roasted fresh orange maize. Besides, from the results on the effect of boiling and roasting on colour properties, the L*, b* and ∆E* values compared positively with the control sample (genotype 8). This observation suggested that the pipeline hybrid genotypes compared well with commercial hybrid used as control. The higher “b*” value has been reported to be an indication of the protein content of the samples^33^. For corn grits hunter colour L*, a* and b* values were found to be 81.5, 2.9 and 23.5, respectively^33^.

### Effects of roasting and harvesting time on bioactive components of fresh orange hybrid maize

Table 2 and Supplementary information S5 showed the results for bioactive components, particularly non-provitamin A carotenoids and other key bioactive constituents of unprocessed fresh orange hybrid maize. In unprocessed fresh orange hybrid maize, xanthophylls (lutein, zeaxanthin, *β*-cryptoxanthin), tannin and vitamin C increased with increases in harvest maturity stages, but phytate content showed a slight decrease at 34DAP. The present results agreed with that reported by Kurilich and Juvik^18^ for β-cryptoxanthin and lutein levels that increased as kernel matured. However, Kurilich and Juvik^18^ found no consistent changes in zeaxanthin, which was contrary to the findings of the present study. The results on phytate content were in close agreement with Horvatic and Balint^16^ that found that phytic acid increased significantly with maturity (p ≤ 0.05).

**Table 2:** Bioactive components of fresh orange hybrid maize at different harvesting time across genotypes and locations (N = 576).

Harvesting time	Bioactive properties^a^					
	^a^Lutein (µ/g)	Zeaxanthin (µ/g)	β-Cryptoxanthin (µ/g)	Phytate (%)	Tannin (%)	Vitamin C (mg/100 g)
**Unprocessed**						
20DAP	6.85c	9.24b	1.99c	2.08b	1.79b	32.6b
27DAP	9.02b	10.6ab	2.83b	2.43a	2.17b	35.1b
34DAP	10.4a	12.5a	4.26a	1.53c	2.72a	44.3a
**Roasted without husk**						
20DAP	4.80b	6.94b	1.87b	1.73b	2.85a	49.0a
27DAP	7.11a	7.91ab	2.70a	2.37a	1.56b	38.4b
34DAP	7.62a	8.58a	2.77a	2.57a	2.76a	49.9a
**Roasted with husk**						
20DAP	8.85b	13.01a	3.11b	0.970c	1.51a	42.3a
27DAP	8.61b	12.6a	3.29b	1.83b	1.61a	26.5b
34DAP	11.5a	12.3a	4.35a	2.54a	1.52a	45.1a
P (location)				***	***	***
P (maturity)	**	***	***	***	***	***
P (method)	***	***	***	***	***	***
P (location × maturity method)	***	***	***	***	***	***
Error	3.92	0.94	0.77	0.16	0.33	38

Mean values with similar letters in the same column do not differ significantly (p > 0.05).

*N* total sample data points.

^a^All bioactive components were determined in duplicate.

*, **, ***Significant at P ≤ 0.05, P ≤ 0.01 and P ≤ 0.001 respectively, *ns* not significant P > 0.05.

Table 2 and Supplementary information S6 and S7 provide the results for bioactive compounds of roasted fresh orange hybrid maize with and without husk respectively. When hybrid maize cobs were roasted without husk, the mean concentrations of all xanthophylls increased with increases in harvest maturity stages. Phytates also increased with harvesting time. At 20DAP, the phytate content in unprocessed fresh orange hybrid maize was higher than that of both roasted fresh orange hybrid maize with and without husks. Esenwah and Ikenebomeh^34^ also agree that phytate content can be lowered by processing. However, recent research has shown that phytates have many health benefits such as antioxidant, anticancer, hypocholesterolemic and hypolipidemic effects^35,36^, while the concentration of tannin and vitamin C that showed a decrease at 20DAP and 27DAP before showing a slight increase at 34DAP. The low level of tannin in the roasted sample could be due to the removal of the condensed form of tannin from the seed by heat. When fresh orange hybrid maize cobs were roasted with husk (Table 2), the mean concentrations of lutein and vitamin C decreased at 27DAP, followed by an increase at 34DAP. *β*-cryptoxanthin and phytate showed a rise in levels across the harvest maturity stages, while zeaxanthin showed a decrease across the harvest maturity stages. Several studies^37,38^ reported that there is an interlink in the biosynthetic pathway of β-cryptoxanthin and zeaxanthin, in which β-cryptoxanthin is a precursor of zeaxanthin. Tannin increased in concentration at 27DAP but decreased at 34DAP. The observed pattern for tannin could be due to the environmental factors, especially the increasing temperature. It has been reported that tannin biosynthesis might be inhibited under the higher temperature of growth. That tannin is polymerized from other polyphenols (catechin and epicatechin), but the mechanism not yet proving^39^.

The combined analysis of variance for the colour and bioactive properties of unprocessed and processed fresh orange maize showed that there was highly significant (P < 0.001) effect for independent variables maturity and method (forms of roasting) for all colour properties of hybrid orange maize. It was observed that there was significant (P < 0.001) three levels of interaction of “location x maturity x method” for the colour and bioactive properties. The present observation suggests that location, maturity and method of processing affect the colour and bioactive properties of hybrid orange maize. It has reported that harvesting time and processing methods affected the carotenoids content of boiled orange maize^9^.

## Conclusions

The present study indicated that non-provitamin A carotenoids (lutein and zeaxanthin), as well as other bioactive ingredients (tannins, phytic acid and ascorbic acid), are present in the biofortified orange maize varieties in considerable quantity. The result showed that in unprocessed biofortified fresh orange maize, lutein, zeaxanthin, tannin and vitamin C increases with an increase in maturity while phytate decrease with increased maturity. Additionally, concentrations of non-provitamin A carotenoids detected in roasted hybrid maize with husk were higher than those roasted without husk. Still, the concentration was lower for phytate, tannin and vitamin C. In conclusion, processing (dry heating) affects the retention of non-provitamin A carotenoids (lutein and zeaxanthin), as well as other bioactive components (tannins, phytic acid and ascorbic acid) and colour properties of the fresh orange maize hybrids.

## Supplementary Information

Supplementary Information
