# Supplementary material for: Harvesting time and roasting effects on colour properties, xanthophylls, phytates, tannins and vitamin C contents of orange maize hybrid
Source: Sci Rep. 2020 Dec 7;10:21327. doi: 10.1038/s41598-020-78433-9 (PMC7721804; doi:10.1038/s41598-020-78433-9)
Supplement: Supplementary file 1 — Supplementary Information [file 41598_2020_78433_MOESM1_ESM.docx]

**Harvesting time and roasting effects on colour properties, xanthophylls, phytates, tannins and vitamin C contents of orange maize hybrid**

Emmanuel O. Alamu^1,2*^, Bussie Maziya-Dixon^2^, Abebe Menkir^2^, Adebayo O. Ogunlade^3^, Olorunfemi Olaofe^4^

^1^Food and Nutrition Sciences Laboratory, International Institute of Tropical Agriculture, Southern Africa Research and Administration Hub (SARAH) Campus PO Box 310142, Chelstone, Lusaka 10101, Zambia;

^2^International Institute of Tropical Agriculture (IITA), PMB 5320, Oyo Road, Ibadan, Oyo State, Nigeria

^3^Center for Healthful Behavior Change, Department of Population Health, New York University Grossman School of Medicine, New York, N.Y., USA

^4^Department of Chemistry, Ekiti State University, Ado-Ekiti, P.M.B.5363, Ekiti State, Nigeria

**Appendix 1: Genotype name of selected orange hybrid maize for the study**

| **s/n** | **Pedigree** | **Source** |
| --- | --- | --- |
| 1 | ACR97TZL-CCOMP1-Y-S3-13-1-B-B-B-B-B-B-B/9450xKI 21-3-2-2-1-3-B-B-B-B-B-B-B-B-B | 09C8073B |
| 2 | (GT-MAS:Gk x BABANGOYO x GT-MAS:Gk)-2-1-3-1-B-B-B-B-B-B-B-B-B-B/(MP420 x 4001 x MP420)-3-1-2-1-B-B-B- | 09C8075B |
| 3 | (KU1409/KU1414-SR/KVI43)-S2-4-1-  BB/4001xB73LPAx4001-33-2-1-B*4 | 09C8087B |
| 4 | (KU1409/KU1414-SR/NC298)-S2-8-1-BB/9450xKI21-1-5-3 2-2-B*5 | 09C8089B |
| 5 | (KU1409/KU1414-SR/NC298)-S2-7-1-BB/9450xKI21-7-3-1-2-4-B*4 | 09C8095B |
| 6 | (KU1409/KU1414-SR/KUI2007)-S2-3-2-BB/9450xKI21-1-5-2-1-2-B*5 | 09C8097B |
| 7 | 9450xKI21-7-2-1-2-B*4/KU1409xMO17LPAxKU1409-27-3-1-1-B*7 | 09C8099B |
| 8 | Oba Super-II ( control) |  |

**Appendix 2: Colour properties of unprocessed fresh orange hybrid maize at different harvesting time across two locations**

| **genotypes** | **maturity** | **^a^ L*** | **A*** | **B*** | **ΔL*** | **ΔA*** | **ΔB*** | **C** | **ΔC** | **ΔE** | **ΔH** |
| --- | --- | --- | --- | --- | --- | --- | --- | --- | --- | --- | --- |
| 1 | 20DAP | 81.6±1.14 | 4.14±0.434 | 32.6±1.67 | -6.64±1.14 | 3.30±0.434 | 26.4±1.67 | 32.8±1.66 | 26.6±1.66 | 27.4±1.50 | 37.6±2.35 |
| 2 | 20DAP | 79.9±3.71 | 3.32±1.10 | 27.1±2.52 | -8.39±3.71 | 2.47±1.10 | 20.9±2.52 | 27.3±2.37 | 21.1±2.37 | 23.0±0.860 | 29.8±3.36 |
| 3 | 20DAP | 79.9±3.14 | 4.19±0.839 | 30.7±3.02 | -8.38±3.14 | 3.35±0.839 | 24.5±3.02 | 31.0±2.88 | 24.7±2.88 | 26.3±1.70 | 35.0±4.07 |
| 4 | 20DAP | 79.7±2.35 | 4.99±1.54 | 30.6±2.25 | -8.56±2.35 | 4.15±1.54 | 24.4±2.25 | 31.0±2.22 | 24.8±2.22 | 26.3±1.90 | 35.0±3.13 |
| 5 | 20DAP | 79.4±2.96 | 4.55±0.907 | 31.6±2.25 | -8.85±2.96 | 3.71±0.907 | 25.4±2.25 | 32.0±2.12 | 25.7±2.12 | 27.3±1.54 | 36.3±2.99 |
| 6 | 20DAP | 79.8±0.460 | 4.52±0.546 | 32.2±3.74 | -8.52±0.460 | 3.68±0.546 | 26.0±3.74 | 32.5±3.76 | 26.3±3.76 | 27.6±3.46 | 37.1±5.32 |
| 7 | 20DAP | 80.5±3.22 | 4.67±0.779 | 32.1±2.33 | -7.82±3.22 | 3.82±0.779 | 25.9±2.33 | 32.4±2.22 | 26.2±2.22 | 27.5±1.22 | 37.0±3.13 |
| 8 | 20DAP | 80.6±2.31 | 3.77±0.867 | 28.5±1.88 | -7.70±2.31 | 2.92±0.867 | 22.3±1.88 | 28.8±1.76 | 22.6±1.76 | 23.9±1.23 | 31.9±2.48 |
| 1 | 27DAP | 79.3±5.53 | 5.97±0.983 | 34.2±5.56 | -8.95±5.53 | 5.13±0.983 | 28.0±5.56 | 34.8±5.36 | 28.5±5.36 | 30.5±2.55 | 40.3±7.56 |
| 2 | 27DAP | 81.4±2.01 | 4.11±0.309 | 34.3±2.54 | -6.87±2.01 | 3.26±0.309 | 28.1±2.54 | 34.6±2.50 | 28.3±2.50 | 29.2±1.93 | 40.0±3.54 |
| 3 | 27DAP | 81.2±1.15 | 5.87±0.820 | 36.8±2.70 | -7.10±1.15 | 5.03±0.820 | 30.6±2.70 | 37.2±2.73 | 31.0±2.73 | 31.8±2.44 | 43.8±3.87 |
| 4 | 27DAP | 80.1±4.01 | 5.88±0.388 | 36.4±4.66 | -8.16±4.01 | 5.04±0.388 | 30.2±4.66 | 36.9±4.57 | 30.7±4.57 | 32.1±2.94 | 43.4±6.46 |
| 5 | 27DAP | 80.3±2.15 | 6.46±1.06 | 37.6±2.68 | -7.97±2.15 | 5.61±1.06 | 31.4±2.68 | 38.2±2.75 | 31.9±2.75 | 33.0±2.19 | 45.1±3.89 |
| 6 | 27DAP | 78.3±5.79 | 6.22±1.33 | 35.7±7.12 | -9.95±5.79 | 5.38±1.33 | 29.5±7.12 | 36.3±7.15 | 30.0±7.15 | 32.4±4.35 | 42.5±10.1 |
| 7 | 27DAP | 81.3±1.33 | 6.23±1.23 | 38.6±3.17 | -6.95±1.33 | 5.38±1.23 | 32.4±3.17 | 39.1±3.32 | 32.9±3.32 | 33.7±3.08 | 46.5±4.69 |
| 8 | 27DAP | 77.9±6.57 | 6.25±0.635 | 34.0±6.94 | -10.4±6.57 | 5.40±0.635 | 27.8±6.94 | 34.5±6.88 | 28.3±6.88 | 31.1±3.28 | 40.0±9.71 |
| 1 | 34DAP | 80.7±1.02 | 6.34±0.401 | 37.9±2.31 | -7.53±1.02 | 5.49±0.401 | 31.7±2.31 | 38.4±2.31 | 32.2±2.31 | 33.1±2.11 | 45.5±3.27 |
| 2 | 34DAP | 79.6±3.18 | 4.80±0.702 | 37.1±1.11 | -8.67±3.18 | 3.95±0.702 | 30.9±1.11 | 37.5±1.18 | 31.2±1.18 | 32.5±1.81 | 44.1±1.67 |
| 3 | 34DAP | 79.1±2.23 | 6.43±0.241 | 38.5±2.23 | -9.19±2.23 | 5.58±0.241 | 32.3±2.23 | 39.0±2.20 | 32.7±2.20 | 34.1±1.48 | 46.3±3.11 |
| 4 | 34DAP | 80.2±1.10 | 5.87±0.979 | 36.8±2.64 | -8.07±1.10 | 5.03±0.979 | 30.6±2.64 | 37.3±2.74 | 31.1±2.74 | 32.1±2.61 | 43.9±3.88 |
| 5 | 34DAP | 79.0±1.29 | 6.94±0.890 | 39.1±2.72 | -9.30±1.29 | 6.10±0.890 | 32.9±2.72 | 39.7±2.82 | 33.5±2.82 | 34.8±2.88 | 47.3±3.99 |
| 6 | 34DAP | 79.6±1.80 | 6.75±1.18 | 38.9±2.03 | -8.72±1.80 | 5.90±1.18 | 32.7±2.03 | 39.4±2.07 | 33.2±2.07 | 34.4±1.65 | 46.9±2.93 |
| 7 | 34DAP | 78.6±0.848 | 7.58±1.34 | 39.7±2.80 | -9.64±0.848 | 6.73±1.34 | 33.5±2.80 | 40.4±2.93 | 34.2±2.93 | 35.6±2.76 | 48.4±4.15 |
| 8 | 34DAP | 80.5±1.73 | 5.66±0.816 | 36.8±0.444 | -7.79±1.73 | 4.81±0.816 | 30.6±0.444 | 37.3±0.398 | 31.0±0.398 | 32.0±0.487 | 43.8±0.563 |

^a^ Parameter means value± SD

| **genotypes** | **maturity** | **^a^ L*** | **A*** | **B*** | **ΔL*** | **ΔA*** | **ΔB*** | **C** | **ΔC** | **ΔE** | **ΔH** |
| --- | --- | --- | --- | --- | --- | --- | --- | --- | --- | --- | --- |
| 1 | 20DAP | 70.7±0.000 | 3.96±0.000 | 25.5±0.000 | -17.5±0.000 | 3.12±0.000 | 19.3±0.000 | 25.8±0.000 | 19.5±0.000 | 26.3±0.000 | 27.6±0.000 |
| 2 | 20DAP | 73.5±3.55 | 2.56±0.803 | 24.1±3.40 | -14.8±3.55 | 1.71±0.803 | 17.9±3.40 | 24.2±3.47 | 18.0±0.409 | 23.7±0.409 | 25.4±4.90 |
| 3 | 20DAP | 71.9±2.62 | 3.37±0.514 | 26.5±4.80 | -16.4±2.62 | 2.52±0.514 | 20.3±4.80 | 26.7±4.83 | 20.4±2.10 | 26.6±2.10 | 28.9±6.83 |
| 4 | 20DAP | 71.7±0.958 | 4.31±0.098 | 25.1±1.42 | -16.6±0.958 | 3.46±0.098 | 18.9±1.42 | 25.5±1.42 | 19.2±0.444 | 25.5±0.444 | 27.2±2.00 |
| 5 | 20DAP | 70.5±0.000 | 5.20±0.000 | 29.3±0.000 | -17.8±0.000 | 4.36±0.000 | 23.1±0.000 | 29.8±0.000 | 23.5±0.000 | 29.5±0.000 | 33.3±0.000 |
| 6 | 20DAP | 69.6±0.087 | 4.23±0.658 | 27.6±1.40 | -18.7±0.087 | 3.39±0.658 | 21.4±1.40 | 28.0±1.49 | 21.7±1.18 | 28.7±1.18 | 30.7±2.10 |
| 7 | 20DAP | 71.2±0.289 | 5.86±0.225 | 30.1±0.150 | -17.1±0.289 | 5.01±0.225 | 23.9±0.150 | 30.7±0.190 | 24.4±0.324 | 29.8±0.324 | 34.5±0.271 |
| 8 | 20DAP | 73.0±0.803 | 3.60±0.618 | 24.0±2.54 | -15.2±0.803 | 2.75±0.618 | 17.8±2.54 | 24.2±2.60 | 18.0±1.46 | 23.6±1.46 | 25.4±3.68 |
| 1 | 27DAP | 72.6±3.53 | 6.92±1.59 | 33.6±5.05 | -15.7±3.53 | 6.07±1.59 | 27.4±5.05 | 34.3±5.26 | 28.1±2.96 | 32.5±2.96 | 39.7±7.44 |
| 2 | 27DAP | 73.5±2.78 | 4.32±0.198 | 30.2±3.42 | -14.8±2.78 | 3.48±0.198 | 24.0±3.42 | 30.5±3.40 | 24.3±1.48 | 28.7±1.48 | 34.3±4.81 |
| 3 | 27DAP | 72.6±3.85 | 6.54±1.91 | 33.8±7.15 | -15.7±3.85 | 5.70±1.91 | 27.6±7.15 | 34.5±7.38 | 28.2±4.72 | 32.8±4.72 | 39.9±10.4 |
| 4 | 27DAP | 72.1±2.37 | 6.03±1.069 | 32.4±3.88 | -16.2±2.37 | 5.19±1.07 | 26.2±3.88 | 32.9±3.81 | 26.7±2.55 | 31.4±2.55 | 37.8±5.38 |
| 5 | 27DAP | 72.1±3.05 | 6.96±3.08 | 33.5±7.09 | -16.1±3.05 | 6.12±3.08 | 27.3±7.09 | 34.2±7.50 | 28.0±5.05 | 32.8±5.05 | 39.6±10.6 |
| 6 | 27DAP | 71.6±4.64 | 7.16±2.27 | 36.1±8.05 | -16.7±4.64 | 6.32±2.27 | 29.9±8.05 | 36.8±8.33 | 30.5±4.88 | 35.5±4.88 | 43.2±11.8 |
| 7 | 27DAP | 72.2±4.43 | 7.23±1.83 | 34.7±7.06 | -16.1±4.43 | 6.39±1.83 | 28.5±7.06 | 35.5±7.23 | 29.2±4.28 | 34.0±4.28 | 41.3±10.2 |
| 8 | 27DAP | 73.4±4.48 | 6.16±2.03 | 32.9±6.71 | -14.9±4.48 | 5.32±2.03 | 26.7±6.71 | 33.5±6.96 | 27.2±4.08 | 31.6±4.08 | 38.5±9.84 |
| 1 | 34DAP | 72.6±2.23 | 7.57±1.29 | 35.1±1.13 | -15.6±2.23 | 6.72±1.29 | 28.9±1.13 | 35.9±1.20 | 29.6±1.07 | 33.6±1.07 | 41.9±1.71 |
| 2 | 34DAP | 72.9±3.40 | 6.38±0.771 | 35.4±2.85 | -15.3±3.40 | 5.53±0.771 | 29.2±2.85 | 36.0±2.88 | 29.7±2.65 | 33.6±2.65 | 42.0±4.07 |
| 3 | 34DAP | 69.6±2.38 | 7.89±1.92 | 33.6±0.9191 | -18.7±2.38 | 7.04±1.92 | 27.4±0.991 | 34.5±1.41 | 28.2±0.85 | 34.0±0.852 | 40.0±2.03 |
| 4 | 34DAP | 69.4±6.17 | 7.16±1.46 | 31.5±5.93 | -18.9±6.17 | 6.31±1.46 | 25.3±5.93 | 32.3±6.10 | 26.1±3.62 | 32.9±3.62 | 36.9±8.62 |
| 5 | 34DAP | 69.6±6.84 | 7.66±1.75 | 36.5±4.77 | -18.7±6.84 | 6.81±1.75 | 30.3±4.77 | 37.3±5.02 | 31.1±1.48 | 37.0±1.48 | 44.0±7.11 |
| 6 | 34DAP | 64.2±10.9 | 7.30±1.19 | 33.8±2.72 | -24.1±10.9 | 6.45±1.19 | 27.6±2.72 | 34.5±2.84 | 28.3±6.04 | 38.1±6.04 | 40.0±4.02 |
| 7 | 34DAP | 69.4±1.03 | 7.98±1.33 | 34.5±1.44 | -18.8±1.03 | 7.13±1.33 | 28.3±1.44 | 35.4±1.58 | 29.1±0.935 | 34.7±0.935 | 41.2±2.24 |
| 8 | 34DAP | 72.0±3.30 | 7.84±2.04 | 32.8±1.31 | -16.3±3.30 | 7.00±2.04 | 26.6±1.31 | 33.8±1.76 | 27.5±0.815 | 32.2±0.815 | 39.0±2.52 |

**Appendix 3:** **Colour properties of roasted fresh orange hybrid maize without husk at different harvesting time across two locations**

^a^ Parameter means value± SD

**Appendix 4: Colour properties of roasted fresh orange hybrid maize with husk at different harvesting times across two locations**

| **genotypes** | **maturity** | **^a^ L*** | **A*** | **B*** | **ΔL*** | **ΔA*** | **ΔB*** | **C** | **ΔC** | **ΔE** | **ΔH** |
| --- | --- | --- | --- | --- | --- | --- | --- | --- | --- | --- | --- |
| 1 | 20DAP | 74.3±5.90 | 7.34±3.22 | 38.4±7.71 | -14.0±5.90 | 6.49±3.22 | 32.2±7.71 | 39.1±8.13 | 32.9±8.13 | 36.6±4.77 | 46.5±11.5 |
| 2 | 20DAP | 41.2±38.4 | 5.14±1.13 | 32.3±7.76 | -47.1±38.4 | 4.29±1.13 | 26.1±7.76 | 32.8±7.45 | 26.6±7.45 | 56.9±33.4 | 37.6±10.5 |
| 3 | 20DAP | 78.4±2.22 | 7.51±1.44 | 41.2±2.46 | -9.87±2.22 | 6.67±1.44 | 35.0±2.46 | 41.9±2.69 | 35.7±2.69 | 37.1±2.77 | 50.4±3.81 |
| 4 | 20DAP | 77.8±2.56 | 7.02±0.704 | 39.6±4.45 | -10.4±2.56 | 6.17±0.704 | 33.4±4.45 | 40.2±4.51 | 33.9±4.51 | 35.6±3.53 | 48.0±6.37 |
| 5 | 20DAP | 76.6±2.82 | 8.39±1.99 | 40.0±5.42 | -11.7±2.82 | 7.54±1.99 | 33.8±5.42 | 40.8±5.61 | 34.6±5.61 | 36.7±4.53 | 48.9±7.93 |
| 6 | 20DAP | 75.3±3.58 | 7.72±1.97 | 41.4±6.20 | -13.0±3.58 | 6.87±1.97 | 35.2±6.20 | 42.2±6.43 | 35.9±6.43 | 38.5±5.23 | 50.8±9.09 |
| 7 | 20DAP | 76.9±1.02 | 9.01±1.24 | 42.4±2.79 | -11.3±1.02 | 8.16±1.24 | 36.2±2.79 | 43.3±2.95 | 37.1±2.95 | 38.8±2.54 | 52.5±4.18 |
| 8 | 20DAP | 76.3±0.953 | 6.32±2.10 | 38.6±7.06 | -12.0±0.953 | 5.47±2.10 | 32.4±7.06 | 39.1±7.30 | 32.9±7.30 | 35.0±7.18 | 46.5±10.3 |
| 1 | 27DAP | 71.2±3.19 | 6.05±0.481 | 32.1±2.05 | -17.0±3.19 | 5.20±0.481 | 25.9±2.05 | 32.7±2.10 | 26.4±2.10 | 31.6±1.13 | 37.4±2.96 |
| 2 | 27DAP | 74.5±3.09 | 4.50±0.605 | 31.3±3.15 | -13.8±3.09 | 3.66±0.605 | 25.1±3.15 | 31.6±3.20 | 25.3±3.20 | 29.1±1.25 | 35.8±4.53 |
| 3 | 27DAP | 70.5±2.18 | 6.38±0.647 | 32.4±1.99 | -17.8±2.18 | 5.54±0.647 | 26.2±1.99 | 33.0±2.05 | 26.8±2.05 | 32.2±1.55 | 37.9±2.89 |
| 4 | 27DAP | 71.3±2.73 | 6.34±0.620 | 31.0±3.63 | -16.9±2.73 | 5.50±0.620 | 24.8±3.63 | 31.6±3.67 | 25.4±3.67 | 30.7±2.47 | 35.9±5.19 |
| 5 | 27DAP | 72.6±4.68 | 6.10±1.53 | 31.9±6.48 | -15.7±4.68 | 5.25±1.53 | 25.7±6.48 | 32.5±6.64 | 26.2±6.64 | 31.2±3.28 | 37.1±9.39 |
| 6 | 27DAP | 72.1±3.67 | 6.51±0.965 | 34.9±2.72 | -16.2±3.67 | 5.67±0.965 | 28.7±2.72 | 35.5±2.84 | 29.2±2.84 | 33.6±1.24 | 41.3±4.02 |
| 7 | 27DAP | 71.7±4.79 | 7.98±1.65 | 34.9±4.13 | -16.5±4.79 | 7.14±1.65 | 28.7±4.13 | 35.8±4.39 | 29.5±4.39 | 34.3±1.70 | 41.8±6.21 |
| 8 | 27DAP | 73.9±2.08 | 5.32±0.439 | 31.9±0.035 | -14.4±2.08 | 4.48±0.439 | 25.7±0.035 | 32.4±0.038 | 26.1±0.038 | 29.8±1.04 | 36.9±0.056 |
| 1 | 34DAP | 69.8±2.66 | 5.30±1.10 | 30.8±3.75 | -18.5±2.66 | 4.45±1.10 | 24.6±3.75 | 31.3±3.87 | 25.0±3.87 | 31.4±1.63 | 35.4±5.48 |
| 2 | 34DAP | 70.0±2.19 | 5.05±0.897 | 33.1±3.16 | -18.3±2.19 | 4.21±0.897 | 26.9±3.16 | 33.5±3.26 | 27.3±3.26 | 33.0±1.44 | 38.6±4.61 |
| 3 | 34DAP | 69.4±3.27 | 6.27±1.32 | 33.7±5.40 | -18.9±3.27 | 5.43±1.32 | 27.5±5.40 | 34.3±5.52 | 28.1±5.52 | 34.2±2.74 | 39.7±7.81 |
| 4 | 34DAP | 70.1±2.61 | 6.64±1.55 | 32.8±4.54 | -18.1±2.61 | 5.79±1.55 | 26.6±4.54 | 33.5±4.75 | 27.2±4.75 | 33.0±2.56 | 38.5±6.73 |
| 5 | 34DAP | 68.0±2.46 | 6.27±1.64 | 31.8±3.63 | -20.3±2.46 | 5.42±1.64 | 25.6±3.63 | 32.5±3.88 | 26.2±3.88 | 33.4±1.66 | 37.1±5.50 |
| 6 | 34DAP | 68.1±2.25 | 6.56±1.20 | 31.6±4.61 | -20.2±2.25 | 5.71±1.20 | 25.4±4.61 | 32.3±4.59 | 26.0±4.59 | 33.1±2.46 | 36.8±6.48 |
| 7 | 34DAP | 68.0±1.55 | 7.41±0.829 | 32.2±4.05 | -20.3±1.55 | 6.57±0.829 | 26.0±4.05 | 33.1±4.08 | 26.8±4.08 | 33.8±2.54 | 37.9±5.77 |
| 8 | 34DAP | 69.1±1.88 | 6.25±1.28 | 31.2±3.29 | -19.2±1.88 | 5.41±1.28 | 25.0±3.29 | 31.8±3.48 | 25.6±3.48 | 32.1±1.66 | 36.2±4.93 |

^a^ Parameter means value± SD

**Appendix 5: Bioactive components of unprocessed fresh orange hybrid maize at different harvesting times across two locations**

| **vGenotypes** | **maturity** | **^a^lutein (µ/g)** | **Zeaxanthin (µ/g)** | **β-cryptoxanthin (µ/g)** | **Phytate(%)** | **Tannin(%)** | **Vit C(mg/100g)** |  |
| --- | --- | --- | --- | --- | --- | --- | --- | --- |
| 1 | 20DAP | 6.86±0.358 | 9.40±2.38 | 1.81±0.455 | 1.55±0.77 | 2.43±0.62 | 60.05±8.26 | |
| 2 | 20DAP | 8.59±1.20 | 8.84±1.32 | 1.95±0.417 | 2.46±0.18 | 2.28±0.32 | 37.64±16.44 | |
| 3 | 20DAP | 8.10±1.17 | 8.54±2.64 | 1.57±0.451 | 1.92±0.17 | 2.40±0.99 | 43.03±20.12 | |
| 4 | 20DAP | 6.04±1.46 | 8.77±2.42 | 2.15±1.14 | 2.47±0.22 | 2.50±0.54 | 39.6±15.12 | |
| 5 | 20DAP | 4.83±0.760 | 7.22±0.915 | 2.12±0.403 | 2.29±0.17 | 2.20±0.71 | 32.69±6.72 | |
| 6 | 20DAP | 9.17±1.75 | 8.22±1.44 | 2.28±0.739 | 2.45±0.29 | 1.93±0.32 | 36.54±15.41 | |
| 7 | 20DAP | 6.15±1.31 | 12.0±3.66 | 2.67±0.438 | 2.50±0.30 | 2.28±0.24 | 37.58±14.92 | |
| 8 | 20DAP | 5.05±0.908 | 10.9±1.91 | 1.34±0.225 | 2.12±0.31 | 2.54±0.98 | 36.64±15.75 | |
| 1 | 27DAP | 10.6±0.925 | 12.0±1.06 | 2.92±0.651 | 1.18±0.22 | 1.03±0.71 | 57.09±9.73 | |
| 2 | 27DAP | 7.95±3.15 | 7.41±3.70 | 1.85±0.727 | 1.45±0.6 | 1.47±1.30 | 51.0±15.46 | |
| 3 | 27DAP | 11.3±3.86 | 10.4±3.06 | 2.47±0.442 | 1.20±0.32 | 0.57±0.62 | 49.76±7.34 | |
| 4 | 27DAP | 7.54±1.53 | 10.0±1.55 | 2.82±1.13 | 1.51±0.33 | 1.42±1.31 | 61.89±13.18 | |
| 5 | 27DAP | 9.06±1.50 | 10.7±2.06 | 3.53±0.515 | 1.44±0.44 | 1.24±0.82 | 57.42±11.68 | |
| 6 | 27DAP | 11.3±1.26 | 8.12±1.73 | 2.83±0.344 | 1.41±0.32 | 1.53±1.26 | 60.12±15.16 | |
| 7 | 27DAP | 7.01±2.17 | 13.3±5.48 | 3.59±1.13 | 1.12±0.30 | 1.44±0.83 | 59.51±5.17 | |
| 8 | 27DAP | 7.37±1.10 | 13.0±5.65 | 2.61±0.582 | 1.4±0.53 | 1.05±0.55 | 51.38±6.01 | |
| 1 | 34DAP | 14.1±3.52 | 14.1±4.73 | 3.60±1.34 | 1.12±0.33 | 1.16±0.85 | 54.55±14.71 | |
| 2 | 34DAP | 9.31±0.749 | 8.18±0.912 | 2.96±1.54 | 1.06±0.21 | 2.42±0.90 | 47.13±7.78 | |
| 3 | 34DAP | 10.4±3.73 | 12.4±4.25 | 3.82±1.35 | 1.17±0.37 | 1.41±0.68 | 46.18±11.84 | |
| 4 | 34DAP | 7.86±2.19 | 9.89±3.33 | 5.19±2.28 | 1.06±0.41 | 1.28±0.73 | 47.55±17.11 | |
| 5 | 34DAP | 9.93±2.61 | 11.6±3.10 | 5.04±1.62 | 1.03±0.42 | 1.50±1.32 | 48.18±11.74 | |
| 6 | 34DAP | 11.5±2.87 | 10.2±3.14 | 4.69±2.53 | 0.89±0.18 | 1.26±0.91 | 46.45±13.7 | |
| 7 | 34DAP | 10.1±1.80 | 16.1±5.04 | 5.21±1.94 | 1.10±0.44 | 1.36±0.74 | 53.4±18.14 | |
| 8 | 34DAP | 10.3±3.42 | 17.2±5.64 | 3.62±0.871 | 1.09±0.22 | 1.19±1.17 | 50.45±13.25 | |

^a^Parameter mean value± SD in dry weight basis

**Appendix 6: Carotenoid components of roasted fresh orange hybrid maize without husk at different harvesting times across two locations**

| **genotypes** | **maturity** | **^a^lutein (µ/g)** | **Zeaxanthin (µ/g)** | **β-cryptoxanthin (µ/g)** | **Phytate(%)** | **Tannin(%)** | **Vit C(mg/100g)** |
| --- | --- | --- | --- | --- | --- | --- | --- |
| 1 | 20DAP | 6.64±0.956 | 10.8±5.05 | 2.19±0.388 | 2.12±0.06 | 1.75±0.03 | 31.2±0 |
| 2 | 20DAP | 3.38±0.519 | 3.71±0.600 | 0.831±0.121 | 2.22±0.10 | 1.85±0.32 | 25.75±2.71 |
| 3 | 20DAP | 3.89±1.60 | 4.58±0.962 | 0.855±0.497 | 1.86±0.10 | 1.79±0.28 | 26.23±2.67 |
| 4 | 20DAP | 4.28±0.699 | 5.29±3.24 | 2.30±0.176 | 2.0±0.00 | 1.52±0.12 | 29.08±3.19 |
| 5 | 20DAP | 5.85±1.81 | 8.67±1.70 | 2.83±0.655 | 2.18±0.10 | 1.81±0.20 | 26.89±2.8 |
| 6 | 20DAP | 6.62±1.68 | 7.22±0.760 | 2.33±0.354 | 2.16±0.10 | 1.25±0.40 | 26.92±3.51 |
| 7 | 20DAP | 5.19±1.11 | 10.6±2.07 | 2.72±0.706 | 2.22±0.07 | 1.26±0.04 | 24.55±0.87 |
| 8 | 20DAP | 2.55±0.780 | 4.67±1.02 | 0.908±0.190 | 2.21±0.24 | 1.95±0.32 | 28.03±5.88 |
| 1 | 27DAP | 6.72±1.65 | 7.81±1.42 | 2.85±0.498 | 1.57±0.56 | 1.05±0.17 | 48.71±9.52 |
| 2 | 27DAP | 4.80±1.40 | 4.28±1.10 | 1.66±0.448 | 1.59±0.58 | 1.13±0.39 | 54.2±11.29 |
| 3 | 27DAP | 6.10±0.548 | 7.14±2.13 | 2.45±0.239 | 1.98±0.87 | 1.09±0.30 | 52.79±13.31 |
| 4 | 27DAP | 7.50±4.25 | 8.00±1.64 | 3.22±0.593 | 1.81±1.01 | 0.94±0.18 | 48.93±4.51 |
| 5 | 27DAP | 9.41±2.97 | 8.09±2.17 | 3.45±0.481 | 1.83±0.45 | 1.28±0.27 | 58.64±11.05 |
| 6 | 27DAP | 9.94±3.73 | 9.30±3.35 | 2.93±1.17 | 1.66±0.95 | 1.17±0.38 | 50.5±9.58 |
| 7 | 27DAP | 6.23±0.168 | 10.5±0.769 | 2.74±0.517 | 1.44±0.37 | 0.88±0.37 | 53.41±2.5 |
| 8 | 27DAP | 6.18±1.34 | 8.18±2.20 | 2.34±0.729 | 1.82±0.82 | 1.17±0.31 | 46.3±9.81 |
| 1 | 34DAP | 9.16±1.84 | 8.30±1.55 | 2.09±0.357 | 1.04±0.09 | 1.48±1.29 | 36.0±3.12 |
| 2 | 34DAP | 7.43±3.29 | 6.48±2.85 | 2.09±0.625 | 0.87±0.07 | 1.56±0.47 | 41.1±1.6 |
| 3 | 34DAP | 6.53±1.26 | 7.85±1.85 | 2.46±0.472 | 1.14±0.11 | 1.38±0.81 | 37.63±2.75 |
| 4 | 34DAP | 7.06±3.62 | 8.73±4.68 | 3.58±1.66 | 1.17±0.1 | 1.78±1.28 | 34.55±2.07 |
| 5 | 34DAP | 9.61±1.61 | 10.2±2.04 | 4.00±0.582 | 1.02±0.08 | 1.64±1.47 | 37.13±2.92 |
| 6 | 34DAP | 8.34±0.905 | 6.99±2.74 | 2.60±0.828 | 0.8±0.08 | 1.14±0.78 | 35.6±2.85 |
| 7 | 34DAP | 6.49±1.83 | 11.0±2.51 | 3.01±1.90 | 1.18±0.25 | 1.87±1.24 | 38.2±0.54 |
| 8 | 34DAP | 6.34±2.02 | 9.18±4.55 | 2.29±1.13 | 0.99±0.28 | 1.47±1.26 | 38.9±3.09 |

^a^Parameter mean value± SD in dry weight basis

**Appendix 7: Carotenoid contents of roasted fresh orange hybrid maize with husk at different harvesting times across two locations**

| **genotypes** | **maturity** | **^a^lutein (µ/g)** | **Zeaxanthin (µ/g)** | **β-cryptoxanthin (µ/g)** | **Phytate(%)** | **Tannin(%)** | **Vit C(mg/100g)** |
| --- | --- | --- | --- | --- | --- | --- | --- |
| 1 | 20DAP | 10.9±0.592 | 14.1±0.874 | 2.79±0.372 | 1.87±1.14 | 1.29±0.83 | 40.21±20.41 |
| 2 | 20DAP | 7.48±0.185 | 8.66±0.238 | 1.65±0.224 | 1.48±0.77 | 0.63±0.37 | 32.9±14.59 |
| 3 | 20DAP | 9.40±2.22 | 10.0±0.255 | 2.72±0.867 | 1.55±0.93 | 0.95±0.81 | 35.52±17.61 |
| 4 | 20DAP | 7.33±0.531 | 8.30±4.01 | 2.28±1.86 | 1.6±0.87 | 1.02±0.43 | 40.01±15.00 |
| 5 | 20DAP | 8.63±1.23 | 14.8±1.72 | 4.80±0.636 | 1.39±0.46 | 0.75±0.54 | 40.67±21.19 |
| 6 | 20DAP | 12.2±1.86 | 12.6±3.43 | 3.40±0.862 | 1.39±0.48 | 1.19±0.72 | 47.07±27.75 |
| 7 | 20DAP | 9.83±1.83 | 21.8±6.37 | 5.25±1.50 | 1.6±0.97 | 1.00±1.01 | 41.78±25.26 |
| 8 | 20DAP | 4.99±2.63 | 13.9±0.390 | 1.97±0.334 | 0.81±0.14 | 0.42±0.19 | 44.1±24.73 |
| 1 | 27DAP | 11.3±1.62 | 12.9±3.93 | 3.00±1.12 | 1.53±0.72 | 1.39±0.21 | 42.92±22.36 |
| 2 | 27DAP | 7.98±1.79 | 11.3±5.51 | 2.55±1.16 | 1.75±0.63 | 1.43±0.56 | 42.17±20.75 |
| 3 | 27DAP | 9.82±1.98 | 13.7±6.92 | 3.54±1.68 | 1.6±0.68 | 1.85±0.36 | 39.86±18.36 |
| 4 | 27DAP | 8.03±2.31 | 12.2±3.13 | 3.96±0.822 | 1.47±0.39 | 1.09±0.08 | 42.17±18.88 |
| 5 | 27DAP | 8.07±2.62 | 13.7±4.55 | 3.69±0.506 | 1.51±0.34 | 1.46±0.18 | 45.97±18.56 |
| 6 | 27DAP | 8.98±0.991 | 10.3±3.55 | 2.97±1.34 | 1.45±0.38 | 1.68±0.85 | 40.45±15.94 |
| 7 | 27DAP | 6.60±0.666 | 11.6±2.81 | 2.99±1.20 | 1.72±0.55 | 1.37±0.27 | 49.16±23.39 |
| 8 | 27DAP | 8.16±2.38 | 15.0±6.29 | 3.60±2.39 | 1.68±0.69 | 1.17±0.04 | 42.7±19.52 |
| 1 | 34DAP | 12.3±1.99 | 13.2±4.99 | 4.53±3.83 | 2.07±1.05 | 0.89±0.83 | 46.45±12.00 |
| 2 | 34DAP | 11.4±2.73 | 10.5±4.88 | 2.55±0.185 | 1.85±0.96 | 1.06±0.69 | 49.85±13.38 |
| 3 | 34DAP | 14.8±5.14 | 12.2±5.17 | 4.82±2.77 | 1.85±0.96 | 0.94±0.5 | 43.15±2.61 |
| 4 | 34DAP | 12.8±8.14 | 11.7±2.71 | 4.71±0.633 | 2.10±1.09 | 1.16±0.67 | 44.13±5.10 |
| 5 | 34DAP | 9.37±2.17 | 11.3±2.48 | 4.68±0.874 | 1.84±0.93 | 1.04±0.47 | 48.93±10.67 |
| 6 | 34DAP | 13.6±4.15 | 10.3±4.24 | 4.73±1.56 | 2.00±0.83 | 1.24±0.6 | 42.1±12.01 |
| 7 | 34DAP | 9.90±1.84 | 17.0±4.67 | 5.73±1.96 | 2.22±0.88 | 1.5±0.93 | 45.6±9.89 |
| 8 | 34DAP | 8.13±1.67 | 12.5±0.680 | 3.05±0.151 | 1.61±0.61 | 0.92±0.76 | 47.9±11.43 |

^a^Parameter mean value± SD in dry weight basis
